# Differential Effects of 3.5 GHz and 24 GHz 5G Radiofrequency Exposure on Male Sexual Behaviour and Reproductive Endocrine Function in Rats

**DOI:** 10.3390/ijms27167102

**Published:** 2026-08-07

**Authors:** Atikah Hairulazam, Siti Fatimah Ibrahim, Khairul Osman, Mohd Helmy Mokhtar, Aini Farzana Zulkefli, Mohd Farisyam Mat Ros, Norazurashima Jamaludin, Syed Muhamad Asyraf Syed Taha, Sivasatyan Vijay, Zahriladha Zakaria, Amyrul Azuan Mohd Bahar, Farah Hanan Fathihah Jaffar

**Affiliations:** 1 Department of Physiology, Faculty of Medicine, Universiti Kebangsaan Malaysia (UKM), Cheras 56000, Kuala Lumpur, Malaysia; 2 Forensic Science Program, Center for Diagnostics, Therapeutics and Investigative Studies (CODTIS), Faculty of Health Sciences, Universiti Kebangsaan Malaysia (UKM), Jalan Raja Muda Abdul Aziz 50300, Kuala Lumpur, Malaysia; 3 Faculty of Electronic and Computer Technology and Engineering (FTKEK), Universiti Teknikal Malaysia Melaka (UTeM), Hang Tuah Jaya, Durian Tunggal 76100, Melaka, Malaysia; 4 Intel Corporation, Jalan Sultan Azlan Shah, Bayan Lepas 11900, Pulau Pinang, Malaysia

**Keywords:** 5G technology, libido, microwave frequency, millimetre-wave frequency, oestrogen, testosterone

## Abstract

The rapid expansion of 5G technology has increased exposure to high-frequency radiofrequency electromagnetic fields (RF-EMF), raising concerns about male reproductive health. This study investigated the effects of 5G frequencies at 3.5 GHz and 24 GHz on male libido, reproductive hormones, the testosterone-to-oestrogen (T/E) ratio, and androgen receptor expression in rats. Eighteen male Sprague–Dawley rats were randomly assigned to Control, 3.5 GHz, and 24 GHz (n = 6/group). Exposed groups received 7 h/day RF-EMF exposure for 60 consecutive days, while the Control underwent sham exposure. During the final week of exposure, mating behaviours were assessed using mount frequency, mount latency, intromission frequency, ejaculation latency, and post-ejaculatory interval. Serum testosterone and oestrogen levels, the T/E ratio and androgen receptor expression in the testis and hypothalamus were evaluated. The 3.5 GHz group demonstrated statistically significantly prolonged ejaculation latency (3480 ± 1576.956, *p* < 0.01, Hedges’ *g* = 1.03) compared with the Control group. In contrast, 24 GHz exposure statistically significantly reduced mount frequency (4.80 ± 0.837, *p* < 0.01, Hedges’ *g* = −0.65) and prolonged the post-ejaculatory interval (588.83 ± 206.41 s, *p* < 0.01, Hedges’ *g* = 1.63) compared with the Control group. Hormonal analysis revealed statistically significant elevation of testosterone levels in the 3.5 GHz group, whereas the 24 GHz group showed statistically significant reductions in testosterone and oestrogen. Androgen receptor expression was unchanged. These findings suggest frequency-dependent effects of 5G RF-EMF on male libido and endocrine balance.

## 1. Introduction

Radiofrequency electromagnetic fields (RF-EMF) generated by modern communication technologies have been studied for their potential effects on biological systems [1,2,3]. One system that has been extensively investigated is the male reproductive system, as the male gonads are considered highly sensitive to RF-EMF exposure [4,5,6,7]. It has been reported that RF-EMF exposure has been associated with changes in sperm parameters, including motility [1], concentration [8], morphology, and DNA integrity [9,10]. While findings from different studies vary due to differences in exposure intensities, duration, and dosimetry techniques, the growing body of evidence points to male fertility as a potentially sensitive endpoint in RF-EMF exposure [11,12].

Most of the previous studies have evaluated the RF-EMF emitted by wireless communication technology that involves low-band (900 MHz) to mid-band frequency (2.45 GHz) [6,13,14,15]. These frequencies represent earlier communication technology adopted in 2G, 3G and 4G. With the rapid transition to fifth-generation (5G) wireless communication technology, the RF-EMF landscape has expanded to include much higher frequencies that were not extensively used in previous communication technology [16]. In addition to the mid-band frequencies such as 3.5 GHz, which fall under microwave RF-EMF, the high-band frequencies introduced in 5G are in the 24–28 GHz range. This new band is called millimetre waves (mmWaves) [17,18].

These high-frequency bands differ fundamentally from microwave frequencies in terms of penetration depth, energy absorption patterns, and therefore, the biological interaction mechanisms [19]. While microwaves can penetrate tissue to a moderate extent, the absorption of mmWaves is limited to the skin surface and superficial biological structures [20,21]. However, research into the biological effects of mmWaves radiation remains limited, creating a pressing need for carefully controlled experimental studies.

Although extensive research has been conducted on the effects of RF-EMF on the male reproductive system, most studies have primarily focused on testicular morphology and sperm quality. In the present study, a different but equally important aspect of male fertility, libido, was evaluated. Despite its critical role in successful reproduction, libido as a behavioural component has received far less attention in RF-EMF research.

Libido is an essential behavioural component of male fertility, which involves a complex process mediated through neural and hormonal mechanisms [22]. In the hormonal aspect, testosterone plays a vital role in maintaining sexual desire, mounting ability, and mating frequency [23,24]. On the same note, oestrogen, which is mainly produced through aromatisation of testosterone, modulates libido, sexual behaviour patterns, and feedback regulation of gonadotropins [25,26]. Alterations in these hormones may therefore translate into changes to sexual performance even when sperm parameters remain unaffected.

Beyond circulating hormone levels, androgen signalling depends critically on androgen receptors (AR) in both the testis and hypothalamus. In the testis, testosterone binding to AR in Sertoli and peritubular myoid cells is essential for spermatogenesis and normal male fertility. Meanwhile, in the hypothalamus and other limbic regions, neuronal AR integrate testosterone to fine-tune male sexual motivation and performance [27,28]. Disruption of AR signalling in these sites impairs spermatogenesis and reduces mating behaviour even when testosterone remains within the normal range, underscoring that libido reflects not only hormone concentrations but also the sensitivity of AR-dependent pathways that couple the hypothalamic–pituitary–gonadal axis to sexual behaviour [27,29].

Understanding how 5G-related frequencies affect libido is essential, as male reproductive behaviour directly dictates mating success and the subsequent reproductive outcomes. Given the global rollout of 5G communication technology, the distinct biophysical characteristics of the frequencies at 3.5 GHz microwave band and 24 GHz mmWaves band may elicit distinct biological reproductive responses. However, comparative studies evaluating the effects of these representative 5G frequencies on male sexual behaviour remain scarce. Moreover, the limited studies investigating RF-EMF-induced changes in sexual behaviour and their associated hormonal regulation have been restricted to frequencies below 2.5 GHz [30]. To better reflect real-life exposure, the present study employed a prolonged daily exposure of 7 h, which was based on the average daily internet usage in Malaysia which is approximately 5 to 8 h [31]. Although this exposure paradigm does not represent a specific occupational setting, it was designed to simulate sustained daily exposure experienced by heavy users of internet-connected devices.

Therefore, the present study aimed to establish an experimental model to compare the effects of two representative 5G frequency bands, namely 3.5 GHz (microwave) and 24 GHz (mmWaves), on male sexual behaviour in Sprague–Dawley rats. In addition to assessing libido, serum testosterone, oestrogen, and AR expression were evaluated to determine whether behavioural alterations were accompanied by endocrine changes. By integrating behavioural and hormonal endpoints, this study provides a more comprehensive understanding of how emerging 5G frequencies may differentially influence male reproductive function beyond conventional assessments of sperm quality.

## 2. Results

### 2.1. Libido Parameter

**Figure 1:**
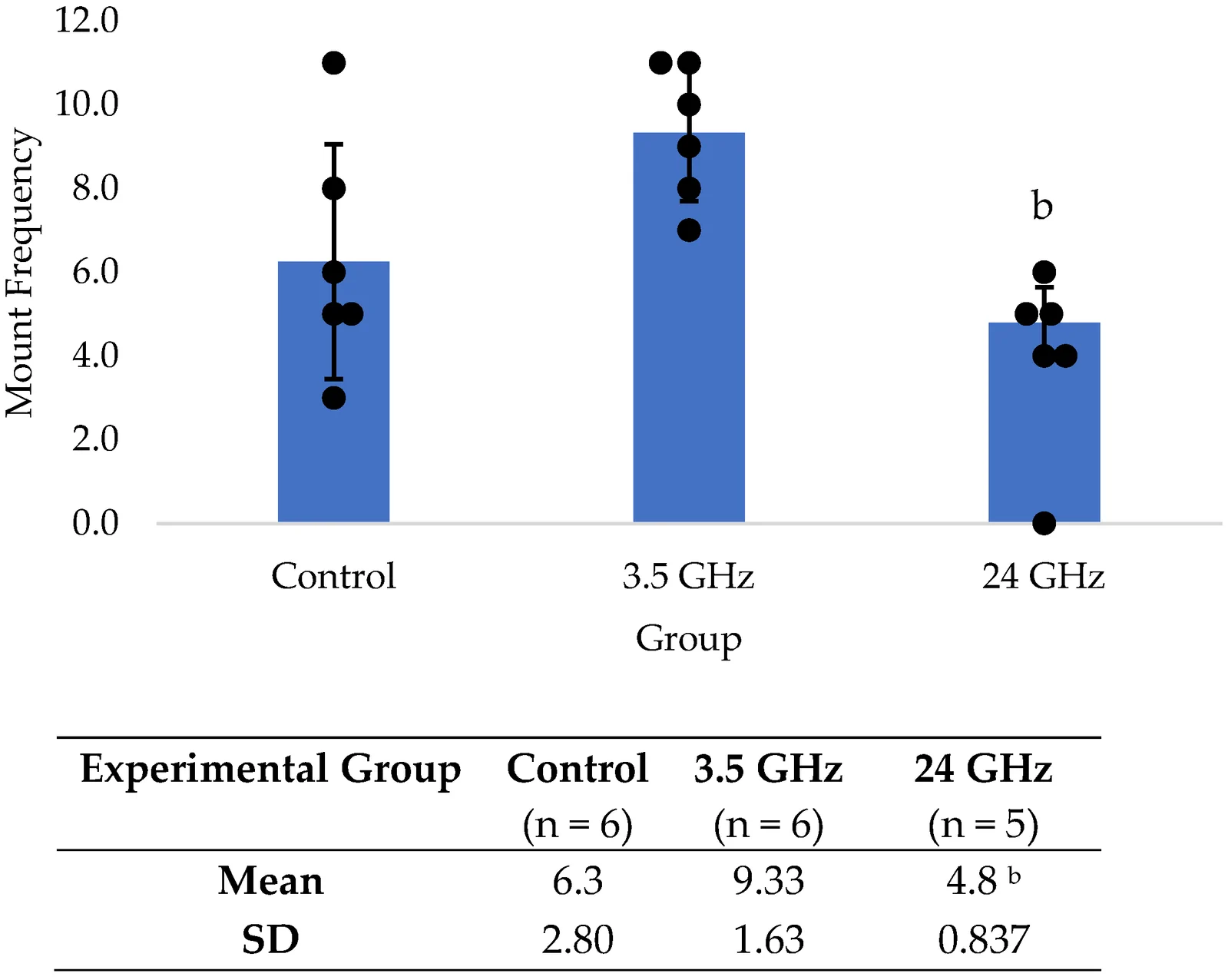
Mount frequency of male rats toward receptive females in each experimental group. Data are presented as mean ± standard deviation (SD). The sample sizes were Control (n = 6), 3.5 GHz (n = 6) and 24 GHz (n = 5). Individual data points represent individual animals. The corresponding group means and SDs are provided in the table below the graph. ^b^ indicates a statistically significant difference (*p* < 0.01, Hedges’ *g* = −3.10) compared with the 3.5 GHz exposure group.

Data analysis demonstrated that the mount frequency (MF) did not show statistically significant differences between the exposed groups and the Control group (Figure 1). However, male rats that received 24 GHz exposure showed a statistically significant reduction in MF compared with 3.5 GHz (*p* < 0.01, Hedges’ *g* = −3.10).

**Figure 2:**
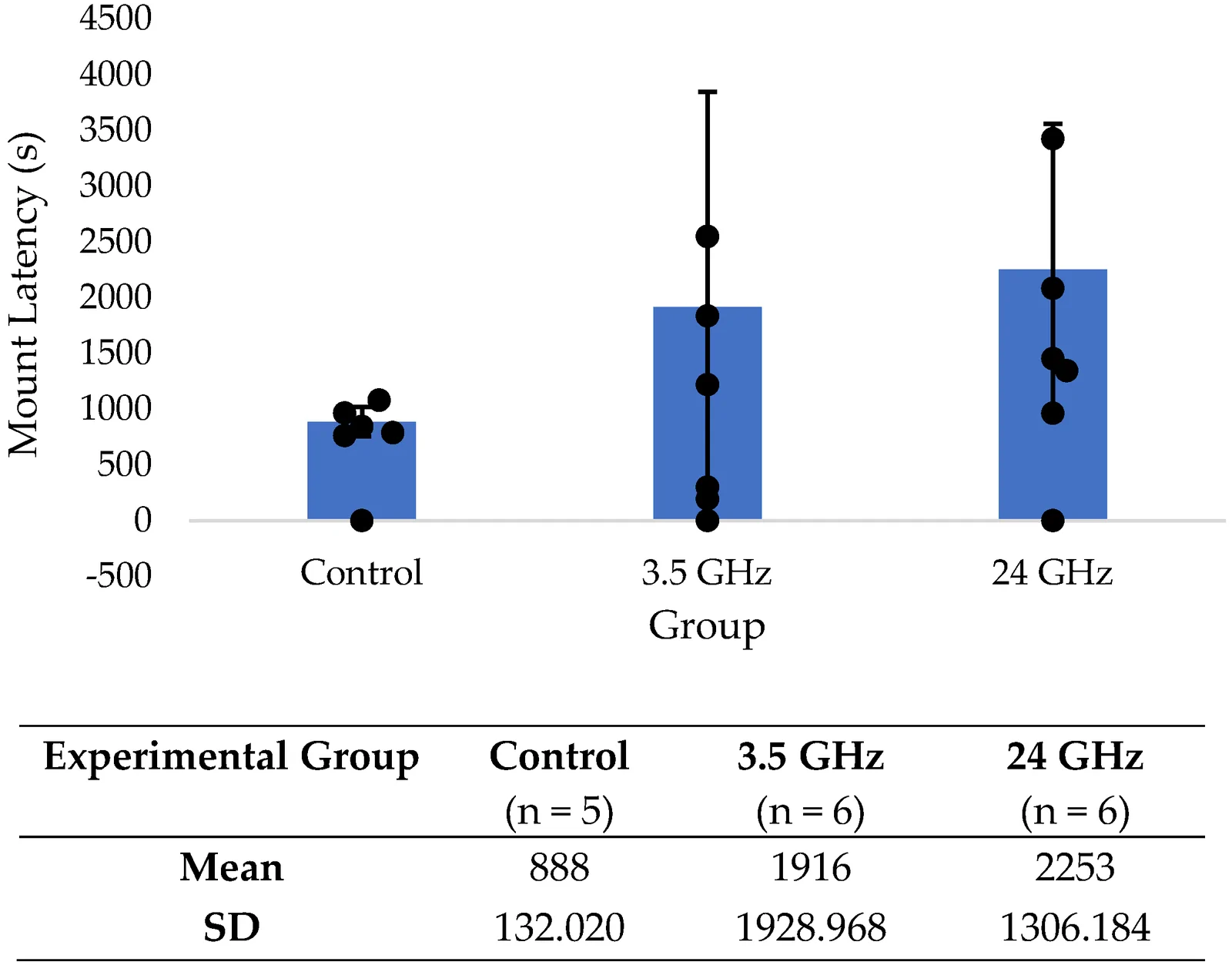
Mount latency of male rats toward receptive females in each experimental group. Data are presented as mean ± SD. The sample sizes were Control (n = 5), 3.5 GHz (n = 6), and 24 GHz (n = 6). Individual data points represent individual animals. The corresponding group means and SDs are provided in the table below the graph. No significant differences among the groups.

**Figure 3:**
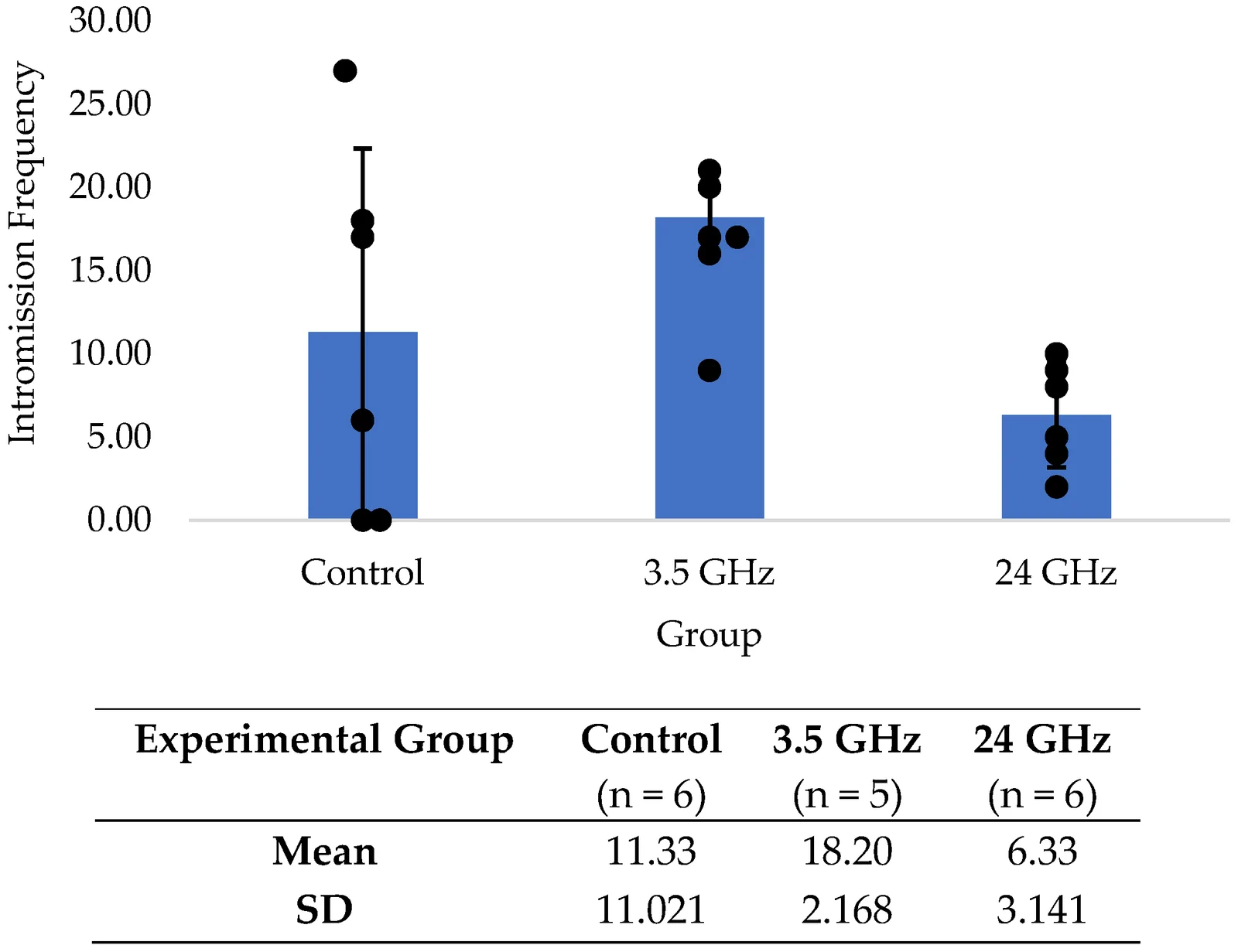
Intromission frequency of male rats toward receptive females in each experimental group. Data are presented as mean ± SD. The sample sizes were Control (n = 6), 3.5 GHz (n = 5) and 24 GHz (n = 6). Individual data points represent individual animals. The corresponding group means and SDs are provided in the table below the graph. No statistically significant differences among the groups.

On the other hand, there were no statistically significant differences in mount latency (ML) (Figure 2) and intromission frequency (IF) (Figure 3) across the groups. However, the 24 GHz group demonstrated a noticeable downward trend relative to the 3.5 GHz-exposed group for both ML and IF.

**Figure 4:**
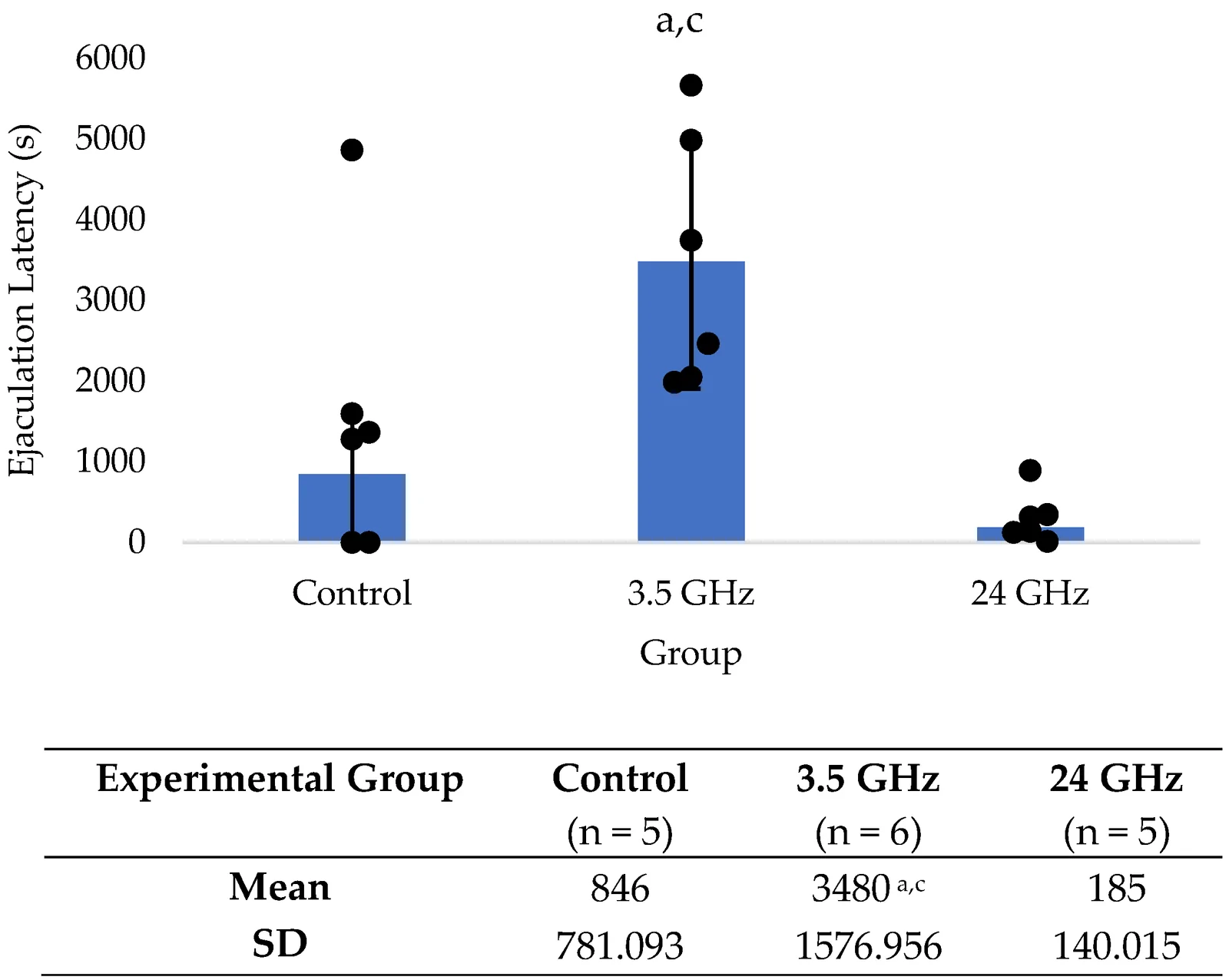
Ejaculation latency of male rats toward receptive females in each experimental group. Data are presented as mean ± SD. The sample sizes were Control (n = 5), 3.5 GHz (n = 6), and 24 GHz (n = 5). Individual data points represent individual animals. The corresponding group means and SDs are provided in the table below the graph. ^a^ indicates a statistically significant difference (*p* < 0.01, Hedges’ *g* = 1.87) compared with the Control group. ^c^ indicates a statistically significant difference (*p* < 0.01, Hedges’ *g* = −2.56) compared with the 24 GHz exposure group.

**Figure 5:**
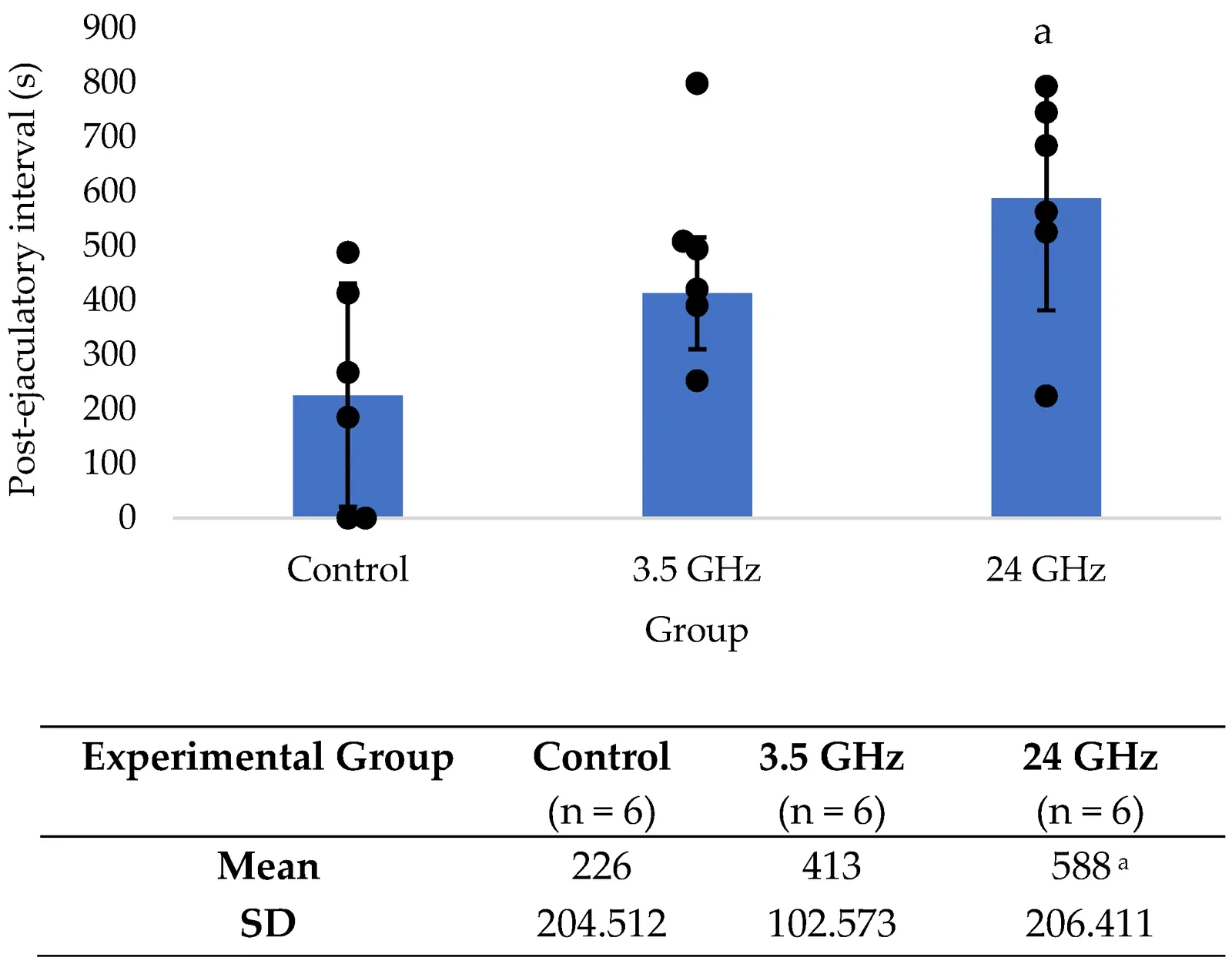
Post-ejaculatory interval of male rats toward receptive females in each experimental group. Data are presented as mean ± SD. The sample sizes were Control (n = 6), 3.5 GHz (n = 5), and 24 GHz (n = 6). Individual data points represent individual animals. The corresponding group means and SDs are provided in the table below the graph. ^a^ indicates a statistically significant difference (*p* < 0.01, Hedges’ *g* = 1.63) compared with the Control group.

The ejaculation latency (EL) was statistically significantly prolonged in the 3.5 GHz group compared with the Control group (*p* < 0.01, Hedges’ *g* = 1.87) (Figure 4). In contrast, the 24 GHz group showed a statistically significant reduction in EL compared with the 3.5 GHz group (*p* < 0.01, Hedges’ *g* = −2.56), indicating an exposure frequency-specific disturbance in ejaculatory timing. Furthermore, post-ejaculatory interval (PEI) was also statistically significantly longer in the 24 GHz-exposed group compared with Control (*p* < 0.01, Hedges’ *g* = 1.63) (Figure 5).

### 2.2. Serum Testosterone, Oestrogen and T/E Ratio

**Figure 6:**
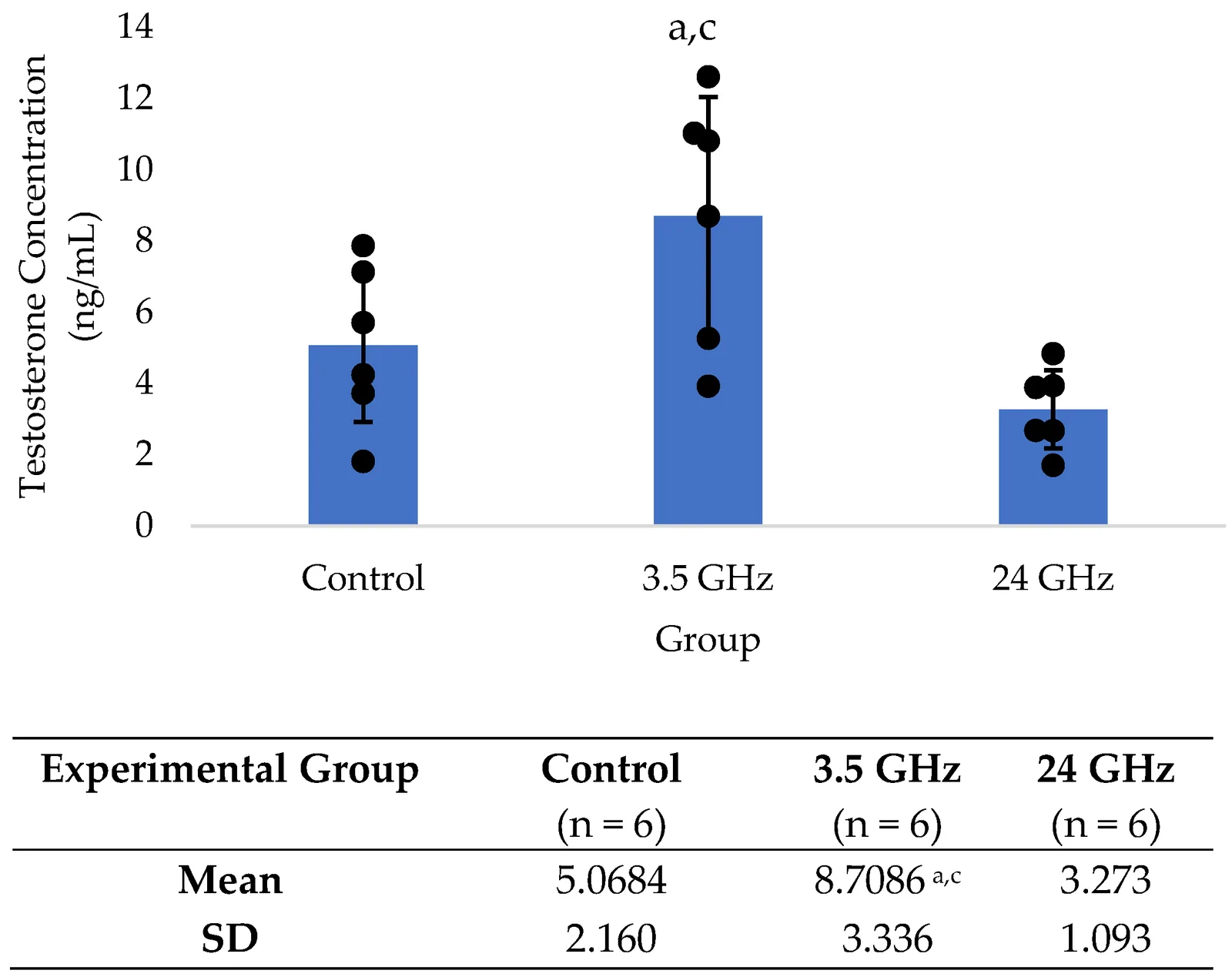
The concentration level of testosterone in serum for each experimental group. Data are presented as mean ± SD (n = 6). Each data point represents the mean value obtained from duplicate measurements of an individual animal. The corresponding group means and SDs are provided in the table below the graph. ^a^ indicates a significant difference (*p* < 0.01, Hedges’ *g* = 1.15) compared with the Control group. ^c^ indicates a statistically significant difference (*p* < 0.01, Hedges’ *g* = −0.27) compared with the 24 GHz exposure group.

There was a statistically significant increase in the levels of serum testosterone in rats exposed to 3.5 GHz, when compared with Control (*p* < 0.01, Hedges’ *g* = 1.15) (Figure 6). However, the testosterone level in the 24 GHz-exposed group fell statistically significantly compared with the 3.5 GHz group (*p* < 0.01, Hedges’ *g* = −1.95).

**Figure 7:**
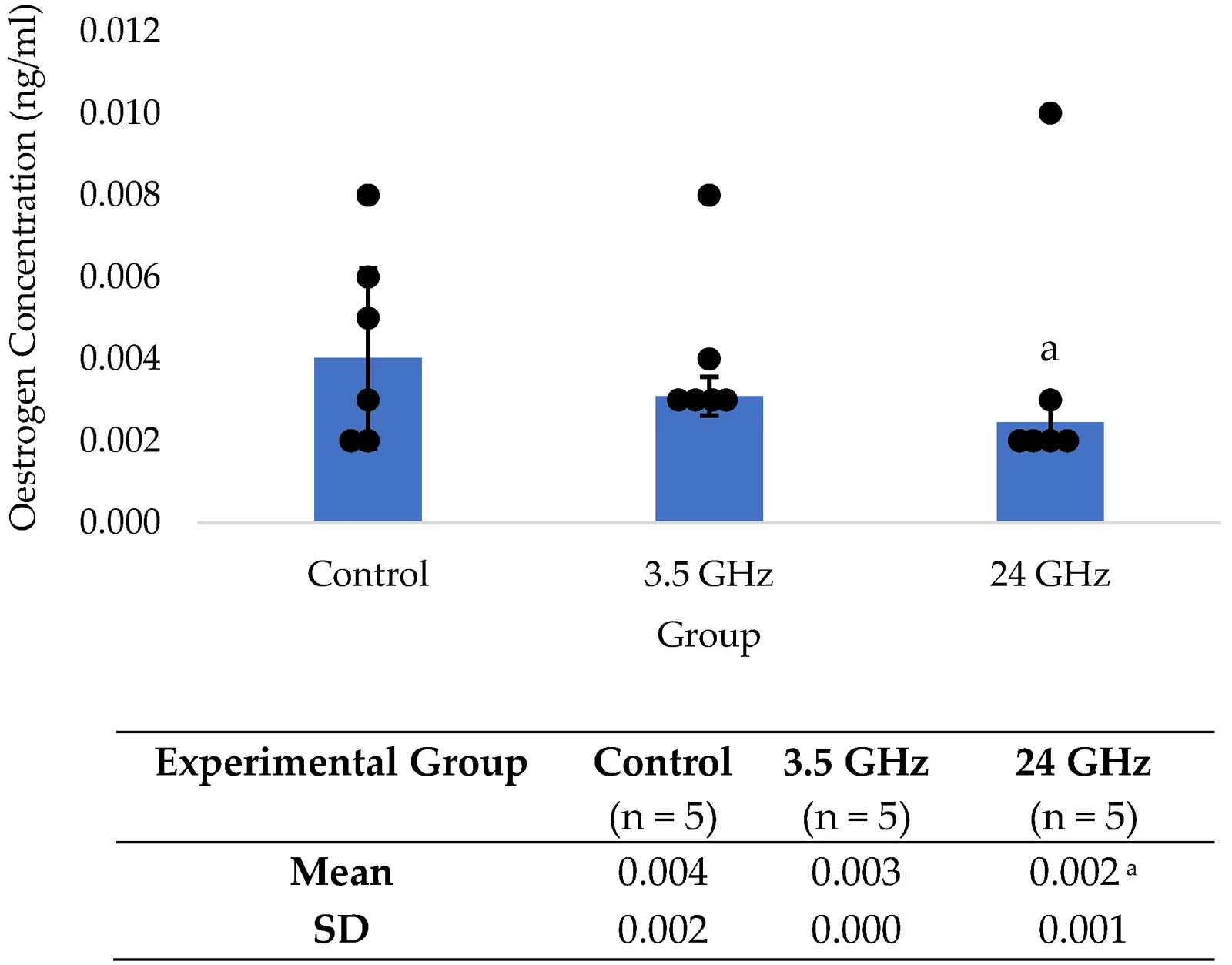
The concentration level of oestrogen in serum for each experimental group. Data are presented as mean ± SD (n = 5). Each data point represents the mean value obtained from duplicate measurements of an individual animal. The corresponding group means and SDs are provided in the table below the graph. ^a^ indicates a statistically significant difference (*p* < 0.01, Hedges’ *g* = −0.27) compared with the Control group.

Similarly, the oestrogen levels of the 24 GHz group showed a statistically significant decrease compared with the Control group (*p* < 0.01, Hedges’ *g* = −0.27) (Figure 7). However, there was no difference in oestrogen between the 3.5 GHz and the Control group.

In addition, the T/E ratio was calculated as total testosterone divided by total oestrogen concentration. As shown in Table 1, rats exposed to 3.5 GHz had a statistically significantly higher T/E ratio (2951 ± 895.51, *p* < 0.05, Hedges’ *g* = 0.72) than the Control group (1764 ± 1449.42). However, this ratio was significantly reduced in the 24 GHz group (1321 ± 554.13, *p* < 0.05, Hedges’ *g* = −1.46) when compared with the 3.5 GHz group.

### 2.3. Expression of the Androgen Receptor

**Figure 8:**
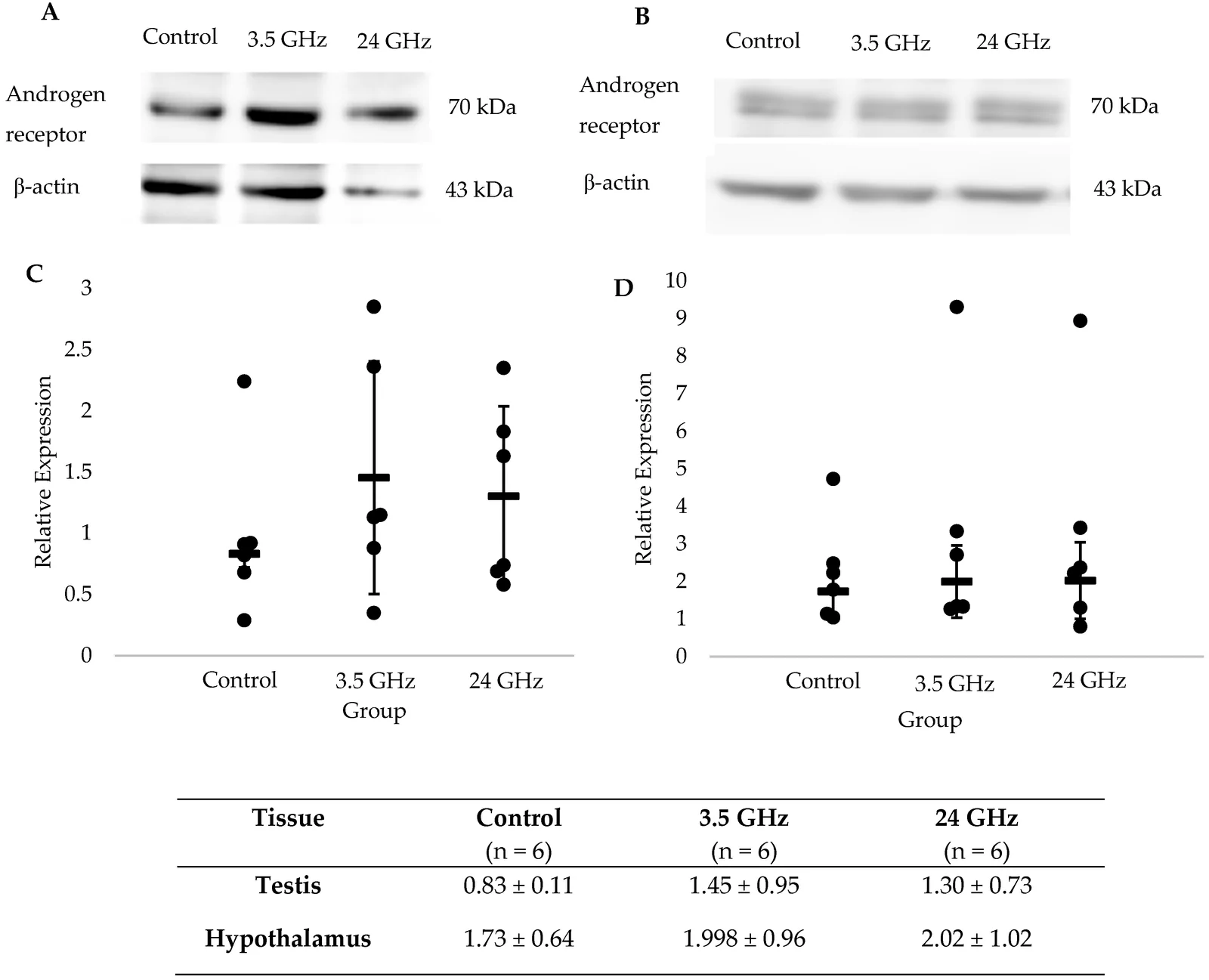
The relative expression of androgen receptors in testis and hypothalamus for each experimental group. Representative immunoblot bands for protein expression levels of AR and β-actin for (**A**) testis tissues and (**B**) hypothalamus tissues. (**C**) The relative AR protein expression normalised to β-actin in the testis. (**D**) The relative AR protein expression normalised to β-actin in the hypothalamus. Individual data points represent values from separate animals. Horizontal lines indicate the group means, and error bars represent the SD (mean ± SD; n = 6 per group). The descriptive mean ± SD values for each tissue and experimental group are shown in the table below the graphs.

Figure 8A,B show the representative Western blot bands for AR expression of each frequency in the testis and hypothalamus, respectively. The results show that there were no statistically significant differences in AR expression in testis tissue between the Control group and both the 3.5 GHz (*p* = 0.426) and 24 GHz (*p* = 0.604) groups (Figure 8C).

For hypothalamic AR expression, densitometric analysis similarly indicated no statistically significant differences in normalised AR protein levels between the Control group and the 3.5 GHz group (*p* = 0.887) or the 24 GHz group (*p* = 0.866) (Figure 8D). Despite there being no significance between groups, a consistent trend toward increased AR relative intensity was observed across the exposure groups.

## 3. Discussion

Consistent with the distinct physical characteristics of the two frequency ranges, RF-EMF exposure in microwave and mmWaves ranges resulted in differential effects on male sexual behaviour and reproductive endocrine profiles. Although both exposed groups underwent the same exposure duration, the behavioural and hormonal responses were not identical. This suggests that the biological effects of RF-EMF may depend on the frequency used, particularly when comparing the mid-band microwave frequency of 3.5 GHz with the newly implemented mmWaves frequency of 24 GHz. However, most assessed parameters remained comparable to the Control group, suggesting that the observed differences were limited to selected behavioural and hormonal endpoints rather than representing widespread alterations in male reproductive function.

In this study, EL was statistically significantly prolonged in the 3.5 GHz group, while MF, ML, and PEI remained comparable to the Control group. EL is primarily governed by supraspinal modulation of the spinal ejaculation generator and depends on the interplay between facilitatory dopaminergic signalling and inhibitory serotonergic pathways within limbic and hypothalamic regions [32,33]. The finding that only EL was altered suggests that exposure to 3.5 GHz radiation did not substantially affect sexual motivation or the initiation of copulatory behaviour. Instead, the prolonged EL may reflect subtle neurophysiological alterations in the central pathways coordinating ejaculation.

This pattern is consistent with a selective alteration of ejaculatory control mechanisms rather than a generalised suppression of male sexual behaviour. However, this interpretation remains speculative given that other behavioural parameters were not significantly altered. As this is among the first studies to investigate the effects of 3.5 GHz and 24 GHz frequencies on male libido parameters, future studies should further explore the neurobiological mechanisms underlying these behavioural changes, particularly the potential involvement of central neurotransmitter systems and neural circuits associated with sexual behaviour and ejaculatory regulation [32,34].

Given that male sexual behaviour is strongly influenced by the balance between testosterone and oestrogen [24,35], alterations in these hormones may contribute to changes in copulatory performance. In the present study, serum testosterone levels were statistically significantly increased in the 3.5 GHz group, whereas oestrogen levels remained comparable to those of the Control group. Interestingly, a similar increase in serum testosterone was observed in our previous study involving chronic exposure to 2.45 GHz radiation, suggesting that prolonged exposure to microwave frequencies may influence endocrine regulation [36].

As a consequence of the elevated testosterone concentration in the absence of a corresponding increase in oestrogen, the testosterone-to-oestrogen (T/E) ratio was also increased in the 3.5 GHz group. The T/E ratio is considered an important indicator of hormonal balance, as both androgenic and oestrogenic signalling contribute to the regulation of male sexual behaviour [23,37]. Therefore, the increase in the T/E ratio suggests a shift in the circulating androgen–oestrogen balance following 3.5 GHz exposure.

Despite these hormonal changes, EL remained statistically significantly prolonged in the 3.5 GHz group. This finding indicates that the increase in testosterone and the T/E ratio did not translate into improved copulatory performance. Furthermore, AR protein expression in both the testis and hypothalamus did not differ statistically significant from the Control group, suggesting that the observed behavioural changes were not accompanied by alterations in receptor abundance. Taken together, these findings suggest that endocrine alterations induced by 3.5 GHz exposure alone may not fully explain the prolongation of EL. As this is among the first studies to investigate the effects of 3.5 GHz exposure on male libido parameters, further studies are warranted to elucidate the neuroendocrine mechanisms linking hormonal changes and sexual behaviour.

In contrast to the 3.5 GHz group, exposure to 24 GHz produced a different pattern of behavioural and endocrine changes. The 24 GHz group showed a statistically significant reduction in MF compared with the 3.5 GHz group, while PEI was statistically significant prolonged compared with the Control group. Meanwhile, ML and IF were not significantly different among the groups. The relatively large within-group variability observed for ML and IF likely reflects the inherent biological differences in sexual behaviour between individual animals. Although all rats were selected based on comparable age, body weight, and health status, and were maintained under identical housing and experimental conditions, individual behavioural responses remain inherently variable in in vivo studies. To improve transparency, all individual animal observations are displayed as overlaid data points in the figures. The reported group means and statistical analyses were performed after outlier assessment, as described in Section 4.8.

Since MF reflects mounting activity and is commonly used as an indicator of sexual motivation, the lower MF observed in the 24 GHz group suggests a reduction in libido-related behaviour compared with the 3.5 GHz-exposed rats. The prolonged PEI further indicates a slower recovery period after ejaculation before the male resumed copulatory activity. Therefore, the behavioural profile of the 24 GHz group suggests a possible alteration in sexual motivation-related behaviour and post-ejaculatory recovery following mmWaves exposure. However, these findings should be interpreted cautiously due to the variability inherent in behavioural assessments and the exploratory nature of the analysis.

The hormonal findings in the 24 GHz group also differed from those observed in the 3.5 GHz group. The testosterone level in the 24 GHz group was statistically significantly lower than in the 3.5 GHz group, while the oestrogen level was statistically significant reduced compared with the Control group. Subsequently, the T/E ratio was statistically significantly lower in the 24 GHz group compared with the 3.5 GHz group. This indicates that 24 GHz exposure did not produce the same testosterone elevation observed after 3.5 GHz exposure. Instead, the reduction in oestrogen and the T/E ratio suggests a different pattern of shift in the androgen–oestrogen balance. Since both testosterone and oestrogen are involved in the regulation of male sexual behaviour, this altered hormonal profile could be attributed to the lower MF and prolonged PEI observed in the 24 GHz group [23,38]. Nevertheless, this interpretation should be made with caution, as the present study assessed only circulating hormone concentrations and did not evaluate steroidogenic enzymes, aromatase activity, or other upstream regulators involved in hormone synthesis and metabolism.

The distinct behavioural and hormonal responses observed between the 3.5 GHz and 24 GHz groups may be partly explained by the different physical characteristics of the two frequencies [19]. The 3.5 GHz frequency belongs to the microwave range and exhibits greater tissue penetration than mmWaves. In contrast, 24 GHz belongs to the mmWaves range, where energy absorption is more superficial and penetration into deeper tissues is more limited [3,20]. These differences in penetration and energy deposition may contribute to the frequency-dependent biological effects observed in the present study. While 3.5 GHz exposure was associated with elevated testosterone levels and prolonged ejaculation latency, 24 GHz exposure was associated with reduced mount frequency, prolonged post-ejaculatory interval, reduced oestrogen levels, and a lower T/E ratio. Collectively, these findings suggest that microwave and mmWaves exposures may be associated with different patterns of behavioural and endocrine responses. Nevertheless, further studies are required to determine whether these changes represent consistent frequency-dependent biological effects.

Despite these behavioural and hormonal alterations, AR protein expression in both the testis and hypothalamus was not significantly altered in the 24 GHz group. Similar findings were also observed in the 3.5 GHz group, indicating that the observed effects were not accompanied by measurable changes in total AR protein abundance. One possible explanation is that RF-EMF exposure under the present conditions influenced circulating hormone levels more readily than receptor expression, which is known to be highly tissue- and cell-specific [27,29]. Furthermore, alterations in hormone availability may affect reproductive behaviour without necessarily producing detectable changes in total AR protein levels within whole-tissue samples. However, because only total AR protein abundance was assessed, no conclusions can be drawn regarding receptor activation, transcriptional activity, or downstream androgen signalling.

In addition, the present study did not evaluate aromatase activity or other regulators of androgen–oestrogen homeostasis, which may have contributed to the hormonal alterations observed following exposure. The use of a 1:1 male-to-female mating ratio may also have limited the sensitivity for detecting subtle changes in male sexual performance. Future studies employing a higher female-to-male ratio and investigating aromatase activity, neurotransmitter regulation, and AR signalling pathways may provide further insight into the mechanisms underlying the observed frequency-dependent effects.

Given the number of behavioural, hormonal, and receptor-related parameters examined in this study, together with the absence of a prespecified primary outcome and correction for multiple comparisons, the findings should be interpreted as exploratory and hypothesis-generating. Therefore, the statistically significant differences observed in selected parameters should be interpreted with caution and require further validation in future studies with larger sample sizes and independent replication. Importantly, most of the assessed behavioural, hormonal, and androgen receptor-related parameters did not show significant differences between groups, suggesting that the observed responses were limited to specific outcomes rather than widespread alterations in male reproductive function. In particular, the behavioural findings involving latency-related parameters should be interpreted cautiously, as these measures may be influenced by inherent individual variability, handling-related factors, and the absence of blinded assessment.

## 4. Materials and Methods

### 4.1. Animals

A total of 36 Sprague–Dawley rats consisting of 18 males (n = 18) and 18 females (n = 18) were obtained from the Laboratory Animal Resources Unit (LARU), Universiti Kebangsaan Malaysia (UKM). In the absence of specific RF-EMF animal size guidelines, sample size was justified using OECD Test Guideline 407, which recommended five males and five females.

Healthy male rats, approximately 8 weeks of age and weighing 200 ± 50 g, with no apparent physical abnormalities or injuries, were used in this study. Exposure commenced when the male rats were approximately 8 weeks old, representing the young adult reproductive stage at which the reproductive organs are functionally mature [38] while minimising potential confounding effects of ageing. Although epididymal sperm is generally present at this age [39], the 60-day exposure period encompassed one complete spermatogenic cycle, ensuring that the sperm evaluated at the end of the experiment had developed entirely under the experimental RF-EMF exposure conditions.

The male rats were obtained one month earlier than the female rats to allow for their exposure to the respective 5G frequency. All animals were housed in individually ventilated cages (IVCs) and kept in the Animal Laboratory, Level 8, Pre-Clinical Building, Faculty of Medicine, UKM. Food pellets and clean water were provided ad libitum under a 12 h light–dark cycle, with the ambient temperature maintained at 22 ± 5 °C.

The male rats were divided randomly into three groups with six rats (n = 6) in each group. The first group was the Control group, which was sham-exposed. The other two groups were 3.5 GHz and 24 GHz, which received exposures at their respective frequencies. The exposure procedure was conducted in the Radiation Room, Level 9 in the Pre-Clinical Building, Faculty of Medicine, UKM. All groups were subjected to the exposure setting for 7 h daily for 60 days.

**Figure 9:**
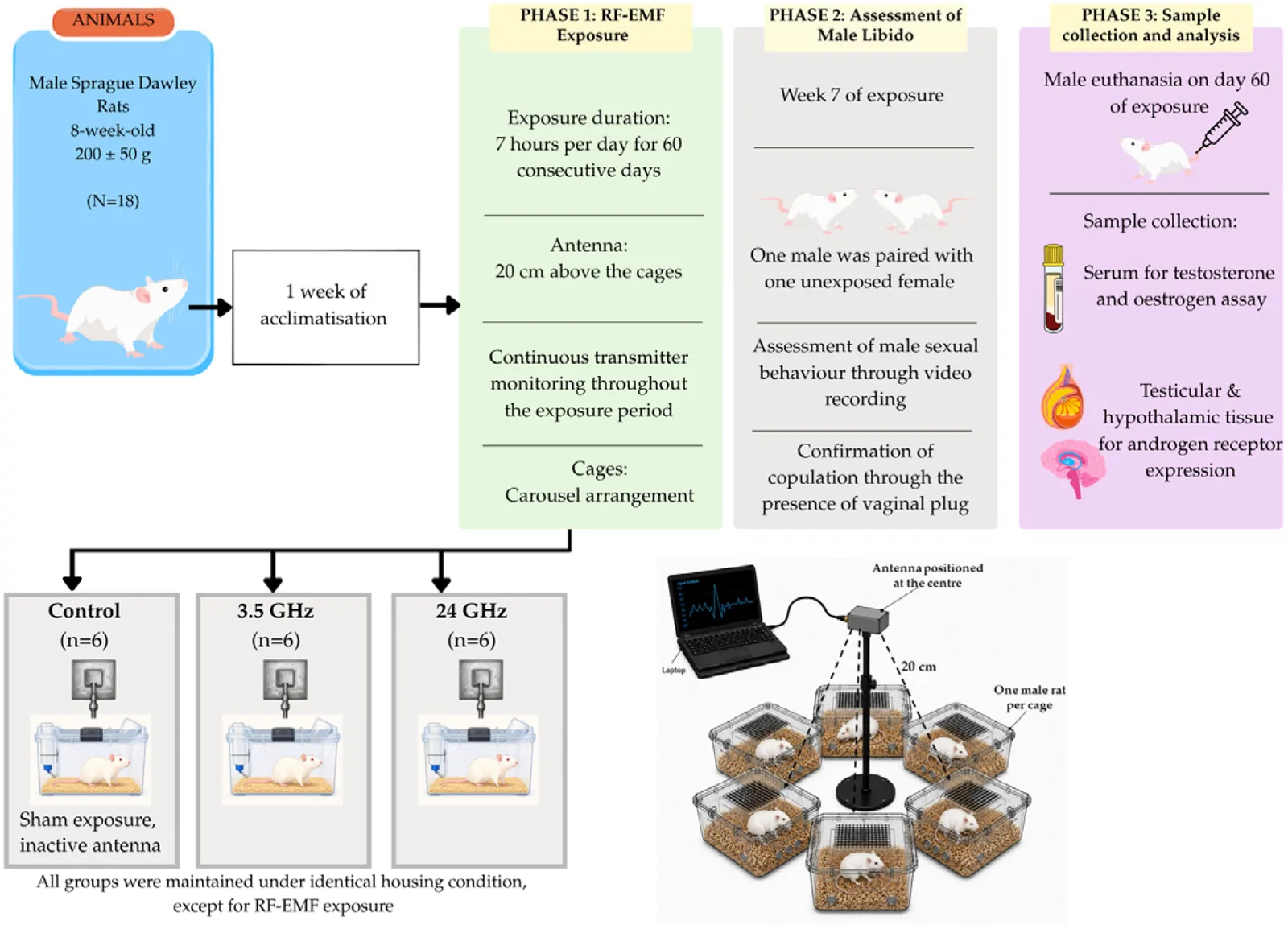
Schematic representation of the experimental workflow. Male Sprague–Dawley rats were acclimatised for one week and then assigned to Control, 3.5 GHz, or 24 GHz groups. RF-EMF exposure was conducted for 7 h/day over 60 days. Male sexual behaviour was assessed by video recording during week 7, followed by sample collection for serum testosterone, oestrogen, and androgen receptor analysis in testicular and hypothalamic tissues.

The body weight of each rat was recorded upon arrival as the baseline measurement and measured weekly throughout the exposure period to ensure no marked changes in general animal health and growth. A schematic overview of the study is shown in Figure 9.

All the procedures involving animals in this study were reviewed and approved by Universiti Kebangsaan Malaysia’s Animal Ethics Committee (UKMAEC) under the ethical approval code FP/2023/FARAH HANAN/24-MAY/1336-SEPT-2023-SEPT-2024.

### 4.2. Exposure Settings

In this study, the exposure model was designed to simulate 5G environmental conditions based on the operating frequencies deployed in public [40,41]. The selected frequencies of 3.5 GHz (mid-band) and 24 GHz (high band) represent the primary spectra allocated for 5G communication, especially in Malaysia [39]. Both frequencies were selected to enable a comparative evaluation of their biological effects, as no prior study has investigated their impact on male libido.

In the previous dosimetric analysis, the simulated SAR values were normalised to 1 W of accepted power at the antenna feed. Numerical simulations were performed at source-to-model distances of 5 and 10 cm, and the corresponding SAR values at the experimental distance of 20 cm were estimated using a local power-law extrapolation [42]. Using the same RF-EMF exposure system, antenna configurations, source-to-cage distance, and carousel cage arrangement, the estimated maximum 10 g averaged SAR and whole-body averaged SAR at the 20 cm exposure distance were 0.15 and 0.06 W/kg, respectively, for the 3.5 GHz exposure. The corresponding values for the 24 GHz exposure were 4.00 × 10^−7^ and 1.80 × 10^−7^ W/kg, respectively [42]. These values were derived from numerical simulations and should not be interpreted as direct SAR measurements in freely moving animals. Accordingly, the estimated SAR values reported here are applicable to the present experimental configuration.

During the experiment, the Control group was also placed in the Radiation Room. However, the antenna or device used for the exposure was set to inactive mode. On the other hand, the two exposed groups were exposed to an active antenna or device. For the 3.5 GHz group, the exposure was done by using an omnidirectional microstrip antenna developed by the Faculty of Electronics and Computer Engineering (FTKEK), Universiti Teknikal Malaysia Melaka (UTeM). The SynthHD signal generator was configured to an output-power setting of 22 dBm. The CST far-field simulation yielded a maximum realised antenna gain of 1.548 dBi at 3.5 GHz. This frequency represents the lower end of the 5G technology spectrum, operating within the microwave radiation range.

In contrast, the exposure for the 24 GHz group utilised a device named the 24 GHz Tuya WiFi Smart Human Presence Detector (Shenzhen, China), which is equipped with an LD2420 24G mmWaves antenna capable of emitting a 24 GHz signal. The manufacturer specifies an operating frequency range of 24.00–24.25 GHz and a maximum equivalent isotropically radiated power (EIRP) of 11 dBm for the HLK-LD2420 module [43].

The nominal incident power density at the central 20 cm reference position was estimated using the far-field free-space relationship described in Recommendation ITU-R BT.1698-1 [44]:

$$S_{inc}\left(r,\theta \right)=\frac{P_{t}G\left(\theta \right)10^{-L_{c}/10}}{4\pi r^{2}}$$

where $P_{t}$ is the power supplied by the transmitter, G(θ) is the antenna gain in the direction of the exposure position, $L_{c}$ is the cable and connector loss in dB and *r* is the distance from the antenna phase centre to the exposure reference position. When EIRP is available, the corresponding relationship is:

$$S_{inc}=\frac{EIRP}{4\pi r^{2}}$$

For 3.5 GHz:

$$\begin{array}{c} P_{3.5}=10^{\left(22-30\right)/10}=0.15849\;W\\ G_{3.5}=10^{1.548/10}=1.428\\ S_{3.5}=\frac{0.15849\times 1.428}{4\pi {\left(0.20\right)}^{2}}=0.4503\;W/m^{2} \end{array}$$

This value is equivalent to 45.03 µW/cm^2^.

For 24 GHz:

$$\begin{array}{c} {EIRP}_{24}=10^{\left(11-30\right)/10}=0.012589\;W\\ S_{24}=\frac{0.012589}{4\pi {\left(0.20\right)}^{2}}=0.02505\;W/m^{2} \end{array}$$

This value is equivalent to 2.505 µW/cm^2^.

**Figure 10:**
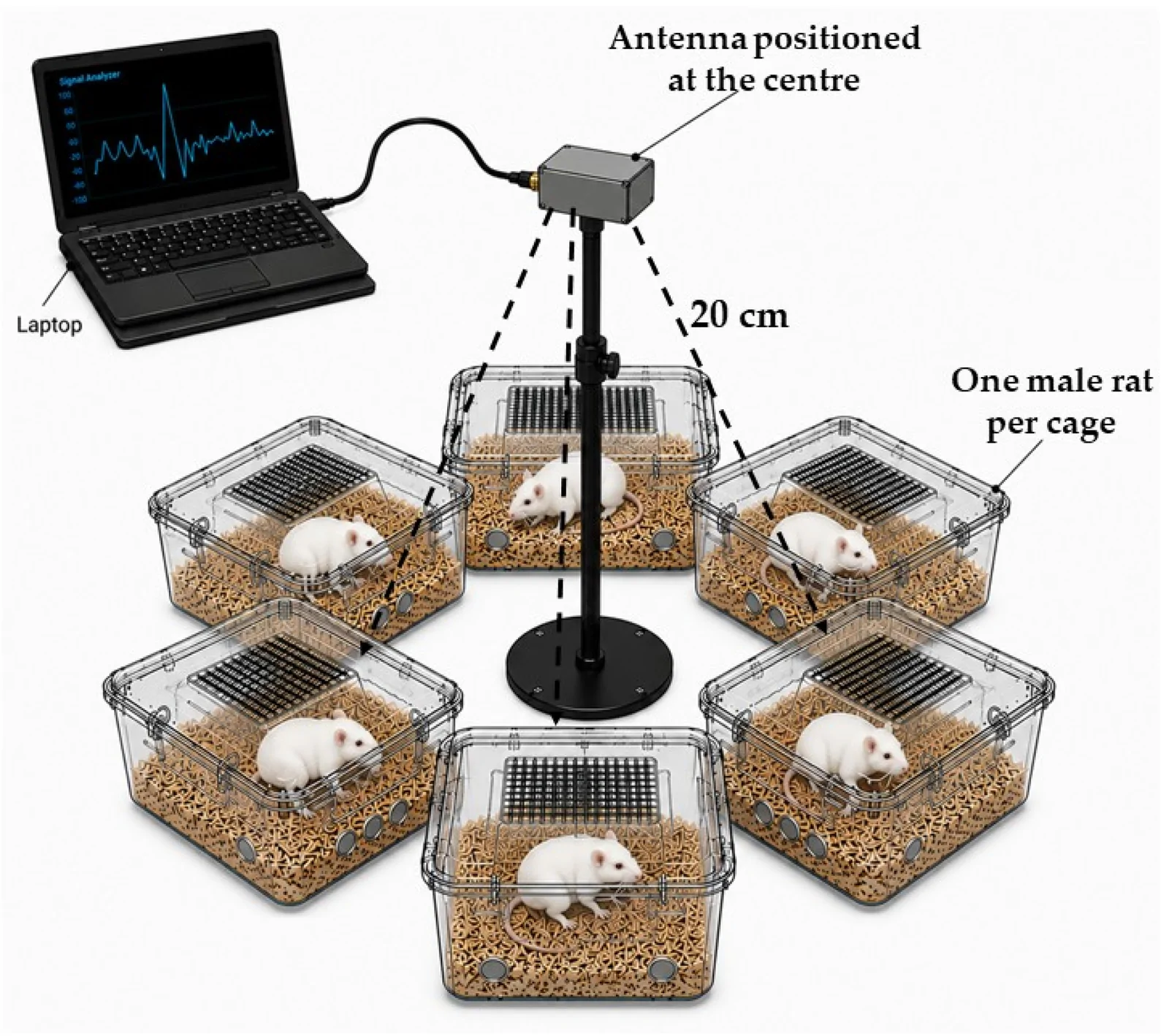
Experimental RF-EMF exposure setup. Six individually ventilated cages (IVCs), each housing one male Sprague–Dawley rat, were arranged symmetrically in a carousel around a centrally positioned antenna. The antenna was connected to a laptop for RF-EMF signal generation and positioned 20 cm above the centre of the carousel arrangement. The sham-exposed Control group underwent the same experimental setup and handling procedures, except that the antenna remained inactive.

The antenna was fixed at the centre of the carousel arrangement and positioned 20 cm above the cage (Figure 10), and the exposure was conducted 7 h per day over 60 consecutive days. The exposure started at 9.00 a.m. and ended at 4.00 p.m. This exposure duration was selected to represent prolonged daily RF-EMF exposure while encompassing one complete spermatogenic cycle in rats [39]. The 20 cm exposure distance was selected based on previous experimental studies investigating the biological effects of RF-EMF [45,46,47]. Furthermore, this distance aligns with international exposure assessment guidelines, including those established by the United States Federal Communications Commission (FCC) and the International Commission on Non-Ionizing Radiation Protection (ICNIRP), which commonly use a 20 cm separation distance to distinguish between close-proximity and far-field/public exposure scenarios [48,49]. This configuration also reflects realistic human exposure conditions, where wireless-emitting devices such as routers, laptops, and fixed antennas are typically located near users rather than in direct contact with the body.

Six individually ventilated cages were arranged symmetrically in a carousel around the centrally positioned antenna. The animals were transferred daily from the holding room to the exposure room for the 7 h exposure session. The cages remained stationary throughout each 7 h exposure session, while the animals were allowed to move freely within their respective cages. Following each exposure session, the animals were returned to the holding room. On subsequent exposure days, there was no fixed assignment of individual animals to specific carousel positions, allowing each animal to occupy different positions throughout the study and thereby reducing systematic position-dependent differences in exposure. The sham-exposed Control group underwent the same transportation, cage arrangement, and handling procedures, except that the antenna remained inactive throughout the exposure period.

For the 3.5 GHz exposure, RF transmission was continuously monitored in real time using a USB-SA124B 12.4 GHz Spectrum Analyzer (Signal Hound, Battle Ground, WA, USA) with Spike software (version 3.9.6) to verify carrier stability and device uptime throughout each session. For the 24 GHz exposure, carrier frequency was verified before and after each session using a Keysight N9010A EXA Signal Analyzer (Keysight Technologies, Santa Rosa, CA, USA), while operational status was monitored throughout the session using the device’s companion application.

However, incident electric-field strength and spatial field uniformity across the individual cage positions were not directly measured. Therefore, the calculated incident power densities represent nominal analytical free-space estimates at the central 20 cm reference position rather than direct measurements at individual cages or animals. For the 3.5 GHz system, the estimate was based on the signal-generator setting and simulated maximum realised gain, whereas for the 24 GHz system it was derived from the manufacturer-specified maximum equivalent isotropically radiated power (EIRP). Accordingly, these values should be regarded as nominal reference estimates for the experimental setup and may not reflect the exact exposure experienced by individual animals.

All metal food holders were removed from the cage and replaced with plastic containers to ensure optimal exposure of the animals to the electromagnetic radiation of the device. This adjustment was necessary as metal is known to interfere by repelling electromagnetic waves [50]. The ambient room temperature was also maintained at approximately 22 ± 5 °C. Before exposure, the EXA Signal Analyzer N9010A was used to verify the presence and carrier frequency of the transmitted signal at the cage position. This verification did not constitute a direct measurement of incident electric-field strength or power density.

### 4.3. Mating Procedure and Evaluation of Libido

Mating was conducted during the seventh week of exposure, commencing one week before the end of the exposure period. Following each daily RF-EMF exposure session, the male rats were returned to their holding cages, and one sexually mature, unexposed female was introduced into each cage on a 1:1 basis to allow natural mating while the male exposure regimen continued throughout the study.

**Figure 11:**
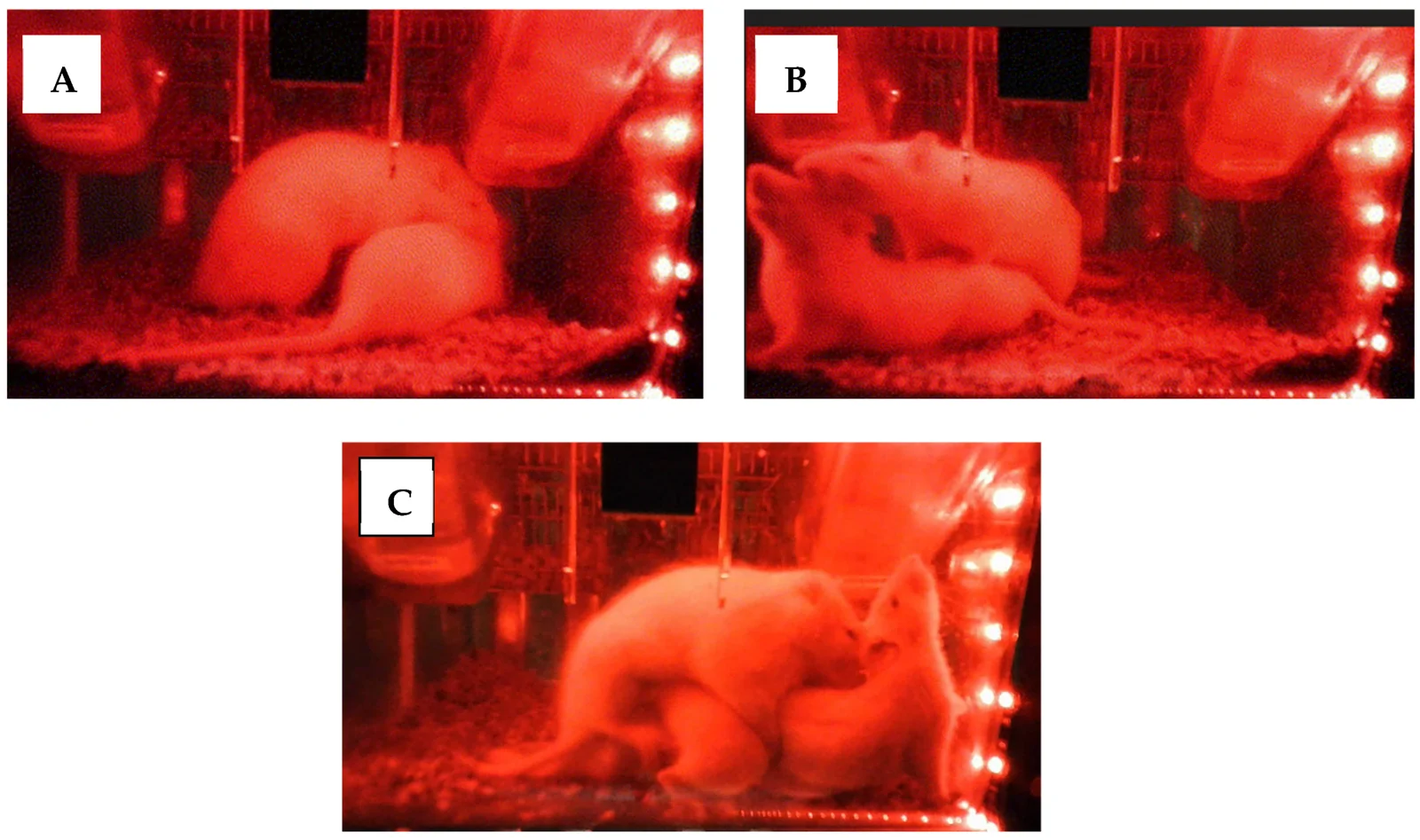
Representative image of mating behaviour in male rats. (**A**) Mounting, (**B**) intromission, and (**C**) ejaculation were identified through manual review of the complete video recordings based on predefined behavioural criteria. Images were captured under low-light red illumination during the mating assessment.

Male sexual behaviour was assessed by manual review of the video recordings by a trained investigator and no automated behavioural-recognition software was used. Mounting, intromission, ejaculation, and post-ejaculatory interval were identified and scored according to the predefined operational criteria described below. Blinding was not implemented during the assessment due to practical and logistical constraints. Representative still images extracted from the video recordings are presented in Figure 11 to illustrate the behavioural events used during manual scoring.

The mating behaviour was continuously recorded from 5:00 p.m. to 4:00 a.m. under low-light conditions by using a mini digital camera (DV007, Shenzhen Yishili Dianzi Shangwu Co., Ltd., Shenzhen, China) mounted to each IVC. Behavioural assessments were conducted according to established protocols described by Agmo (1997) [51], Yakubu & Akanji (2011) [52] and Zang et al. (2016) [30]. The following male sexual behaviour indices were documented during the observation procedure:

1. Mount frequency (MF): the total number of mounting attempts in which the male positioned his forequarters over the female’s hind region without achieving vaginal penetration.
2. Mount latency (ML): the time interval between the initial introduction of the female until the first mount.
3. Intromission frequency (IF): the number of successful vaginal penetrations made by the male.
4. Ejaculation latency (EL): the time interval from first intromission until ejaculation.
5. Post-ejaculatory interval (PEI): the duration from ejaculation until the next intromission.

To distinguish mounting, intromission, and ejaculation during the assessment of male rat sexual behaviour, the video recordings were carefully reviewed to identify each behavioural phase. Mounting was defined as the male positioning himself over the female without penile insertion [32] (Figure 11A). This behaviour usually occurred repeatedly before intromission or ejaculation and was short in duration. Intromission was identified when the male achieved penile insertion into the female vagina, accompanied by a deeper pelvic thrust lasting approximately 200–300 ms (Figure 11B). Following intromission, the male typically performed several pelvic thrusts and subsequently engaged in genital grooming [32]. Ejaculation was identified after a series of successful intromissions and was characterised by a deep, prolonged pelvic thrust associated with semen release (Figure 11C). After ejaculation, the male commonly engaged in self-grooming and entered a period of sexual inactivity, referred to as the post-ejaculatory interval [32,53].

In addition to the behavioural assessment, each female rat was examined manually for the presence of a vaginal plug on the following morning to confirm successful copulation. Upon detection of a vaginal plug, the mating procedure was terminated.

### 4.4. Animal Euthanisation and Blood Sampling

After all the mating procedures were done, the male rats were anaesthetised with a single intraperitoneal injection of an overdose of a Ketamine (3.34 mg/kg, Troy Laboratories Pty Ltd., Glendenning, New South Wales, Australia), Xylazine (3.34 mg/kg, Troy Laboratories Pty Ltd., Glendenning, New South Wales, Australia) and Zoletil-50 (1.66 mg/kg, Virbac, Milperra, New South Wales, Australia) (KTX) cocktail. The death of the animals was confirmed by the loss of right reflex, loss of tail pinch reflex, loss of forelimb and hindlimb pedal reflex and absence of corneal reflex. Blood samples were collected immediately following euthanasia using the cardiac puncture technique. The blood was drawn into BD Vacutainer SST II Advance Plus blood collection tubes (BD, Franklin Lakes, NJ, USA), as described by Jaffar et al. (2022) [47].

The samples were allowed to clot at room temperature for 30 min, and subsequently centrifuged at 12,000 rpm for 10 min at 4 °C to separate the serum. The obtained serum was carefully aliquoted and stored at −80 °C until further analysis using an enzyme-linked immunosorbent assay (ELISA) for hormone quantification.

### 4.5. Determination of Testosterone Concentration

Serum testosterone levels were quantified using a competitive ELISA kit (E-OSEL-R003; Elabscience Biotechnology Co., Ltd., Wuhan, China), following the manufacturer’s protocol. Briefly, the assay components including wash buffer, standards, and horseradish peroxidase (HRP) conjugate were prepared immediately prior to testing. A standard curve ranging from 10 ng/mL to 0.16 ng/mL was generated through serial dilution of the supplied testosterone standard.

A total of 50 μL of serum sample and standard was added to each well of a 96-well microplate precoated with anti-testosterone antibody in duplicate. Subsequently, 50 μL of HRP conjugate was dispensed into each well, and the plate was sealed and incubated for 60 min at 37 °C. After the incubation, wells were aspirated and washed five times with the wash buffer to remove unbound materials. Next, 90 μL of 3′,5,5′-tetramethylbenzidine (TMB) substrate solution was added to each well and allowed to react for 15 min at 37 °C in the dark. The reaction was terminated by adding 50 μL stop solution. The optical density was measured immediately at 450 nm using a microplate reader (SpectraMax Plus 384, Molecular Devices, San Jose, CA, USA). Testosterone concentrations were calculated from the standard curve with a correlation coefficient of r = 0.996 using MyAssay (Brighton, East Sussex, UK) https://myassays.com/, accessed on 15 July 2026.

### 4.6. Determination of Oestrogen Concentration

Serum oestrogen concentrations were determined using a competitive ELISA kit (E-EL-0152; Elabscience Biotechnology Co., Ltd., Wuhan, China), following the manufacturer’s instructions. All reagents, including wash buffer, standards, biotin-labelled detection antibody, and HRP conjugate, were freshly prepared before use. A serial dilution of the supplied oestrogen standard was performed to establish a standard curve ranging from 100 pg/mL to 1.56 pg/mL.

For the assay, 50 μL of serum samples or standards were added in duplicate into wells of a 96-well microplate precoated with anti-oestrogen antibody. Then, 50 μL of biotin-conjugated detection antibody was added to each well, and the plate was incubated for 45 min at 37 °C. After incubation, wells were aspirated and washed three times using the wash buffer to remove unbound reagents. Subsequently, 100 μL of HRP working solution was added to each well, followed by 30 min of incubation at 37 °C. Wells were then washed five times, and 90 μL of TMB substrate was added. The plate was incubated for 15 min at 37 °C in the dark to allow colour development.

The reaction was stopped with 50 μL stop solution, and the optical density was immediately measured at 450 nm using a microplate reader (SpectraMax Plus 384, Molecular Devices, San Jose, CA, USA). Oestrogen concentrations were calculated using a standard curve with a correlation coefficient of r = 0.994, and all sample values were determined using MyAssay.

### 4.7. Assessment of Androgen Receptors in the Testis and Hypothalamus

#### 4.7.1. Extraction of the Testis Tissue

The testes were carefully dissected from each male rat after euthanasia. Any residual blood was thoroughly removed using pre-warmed Phosphate-Buffered Saline (PBS) (Oxoid Ltd, Basingstoke, Hampshire, UK). Then, the testes were immersed in liquid nitrogen and stored at −80 °C for further analysis.

#### 4.7.2. Extraction of the Hypothalamus Tissue

The skull was carefully opened to remove the whole brain. The hypothalamus region was identified according to the atlas of Paxinos and Watson [54]. The brain was positioned with the ventral surface facing up, and a 4 mm thick slice containing the hypothalamus was dissected. The tissues were immediately snap frozen in liquid nitrogen and stored at −80 °C.

#### 4.7.3. Protein Extraction and Quantification

The testis and hypothalamus tissues were rinsed with ice-cold PBS to remove residual blood. For testicular protein extraction, 0.3 g of the testicular tissue was minced on ice and lysed in 1 mL Radioimmunoprecipitation Assay (RIPA) lysis buffer (tissue:RIPA, 1:10) freshly supplemented with 10 µL of phenylmethylsulfonyl fluoride (PMSF) (100 mM) and sodium orthovanadate (Na_3_VO_4_) (100 mM). For the hypothalamus protein extraction, 0.03 g of dissected hypothalamic tissue was lysed in 300 µL RIPA lysis buffer (1:10) freshly supplemented with 3 µL of PMSF (100 mM) and Na_3_VO_4_ (100 mM). Tissues were homogenised using a needle-and-syringe method on ice, incubated for 30 min on ice with orbital shaking, then centrifuged at 12,000 rpm, 4 °C (testis: 10 min; hypothalamus: 15 min). Supernatants were collected as total protein lysates, aliquoted, and stored at −80 °C. Then, the protein content of the samples was determined using a bicinchoninic acid (BCA) protein assay (Thermo Scientific, Rockford, IL, USA) with Bovine Serum Albumin (BSA) (Thermo Scientific, Rockford, IL, USA) standard prepared for a standard curve. The correlation coefficients for both testicular protein and hypothalamus protein are r = 0.999 and 0.0997, respectively.

#### 4.7.4. Sodium Dodecyl Sulphate–Polyacrylamide Gel Electrophoresis (SDS-PAGE)

Protein samples were separated using an SDS-PAGE gel kit (Elabscience Biotechnology Co., Ltd, Wuhan, China) with 5% stacking gel and 10% resolving gel. For each sample, 50 µg of total protein was mixed with 5× SDS loading buffer to make a final volume of 10 µL. The solution was then denatured at 90 °C for 5 min and briefly centrifuged at 12,000 rpm for 30 s. Electrophoresis was run at 80 V for 10 min for the stacking gel and 150 V for 45 min for the resolving gel. The protein was then transferred to 0.45 µm polyvinylidene difluoride (PVDF) membranes (Elabscience Biotechnology Co., Ltd, Wuhan, China) using a wet transfer system. PVDF was activated in methanol for 1 min before assembly, and transfer was conducted in transfer buffer containing methanol with ice block at 40 V for 2 h.

#### 4.7.5. Immunodetection of Androgen Receptors

The membrane was washed briefly in TBST (Elabscience Biotechnology Co., Ltd, Wuhan, China), blocked in 3% skim milk in TBST for 1 h at room temperature. Then, the membrane was incubated with an androgen receptor (AR) polyclonal antibody (Elabscience Biotechnology Co., Ltd, Wuhan, China, 1:500) in 3% skim milk overnight at 4 °C. After the membrane was washed using TBST 3 times for 15 min, the membrane was incubated with goat anti-rabbit IgG (H+L), HRP conjugated (1:2000) for 1 h at room temperature, and washed with TBST (3 × 15 min). Bands were visualised using an Enhanced Chemiluminescence (ECL) substrate kit (Elabscience Biotechnology Co., Ltd, Wuhan, China) and imaged using a chemiluminescence imager.

#### 4.7.6. Stripping and Re-Probing for Housekeeping Control

After AR detection, the membrane was washed in TBST and incubated with stripping buffer for 30 min at room temperature. Then, the membrane was washed using TBST prior to re-probing with a housekeeping protein antibody on the same membrane using a primary antibody, rabbit polyclonal beta-actin antibody (Elabscience Biotechnology Co., Ltd, Wuhan, China), overnight. Then, the membrane was incubated with a secondary antibody, mouse monoclonal (AC-15) beta-actin (HRP) (Abcam, Cambridge, UK), for 1 h at room temperature. Bands were visualised using a chemiluminescent substrate (ECL) kit (Elabscience Biotechnology Co., Ltd, Wuhan, China) and imaged using a chemiluminescence imager. Optical densities of the protein bands were analysed using ImageJ software (version 1.54g). The results were expressed as a relative of the target protein/β-actin ratio.

### 4.8. Statistical Analysis

All results were expressed as mean ± standard deviation (mean ± SD). The normality of data distribution was assessed using the Shapiro–Wilk test. Homogeneity of variance was evaluated using Levene’s test. Outliers were screened using the SPSS boxplot procedure and cross-checked using Tukey’s interquartile range criterion, where values below Q1 − 1.5 × IQR or above Q3 + 1.5 × IQR were identified as statistical outliers. For normally distributed data with equal variances, comparisons among the Control, 3.5 GHz and 24 GHz groups were performed using one-way analysis of variance (ANOVA), followed by Tukey’s post hoc test for multiple comparisons. One-way ANOVA was used to compare the significant differences in libido, sperm parameters and hormone profiles between groups. A *p*-value < 0.05 was considered statistically significant. Pairwise effect sizes were calculated using bias-corrected Hedges’ *g* for independent groups. Statistical analysis was performed using SPSS version 13.0 (SPSS Inc., Chicago, IL, USA).

## 5. Conclusions

In conclusion, chronic exposure to 5G-related RF-EMF at 3.5 GHz and 24 GHz produced distinct alterations in male sexual behaviour, accompanied by different hormonal profiles. Exposure to 3.5 GHz was associated with elevated testosterone levels and prolonged ejaculation latency. On the other hand, exposure to 24 GHz was associated with reduced mounting activity, prolonged post-ejaculatory recovery, decreased oestrogen levels, and a lower T/E ratio. These findings suggest that different 5G frequencies may influence male reproductive function through distinct biological pathways rather than producing a uniform response. Although 24 GHz mmWaves exposure is generally characterised by superficial energy absorption and limited tissue penetration, the present findings demonstrate that measurable behavioural and endocrine alterations may still occur following chronic exposure under the conditions examined. While these results do not establish causality or indicate adverse reproductive outcomes in humans, they highlight the need for continued investigation of the biological effects of mmWaves frequencies, particularly as their use in wireless communication technologies continues to expand.

However, the current findings represent an exploratory investigation, particularly given the introduction of newer RF-EMF frequencies operating at different wavelengths and their potential biological interactions. Therefore, the observed changes should be interpreted cautiously and considered as preliminary observations that may provide a basis for future studies investigating the effects of emerging RF-EMF technologies on male reproductive behaviour and their underlying physiological mechanisms. Further investigations incorporating different exposure parameters, larger sample sizes, additional mechanistic endpoints, and refined experimental approaches will be necessary before definitive conclusions can be drawn regarding the potential effects of RF-EMF exposure on male sexual behaviour and reproductive function.

## Figures and Tables

**Table 1:** The T/E ratio of male rats in each experimental group. Data are presented as mean ± SD (n = 6).

	Control (n = 6)	3.5 GHz (n = 6)	24 GHz (n = 6)
T/E ratio	1765 ± 1449.42	2951 ± 895.51 ^a^	1321 ± 554.13 ^b^

^a^ indicates a statistically significant difference (*p* < 0.05, Hedges’ *g* = 0.72) compared with the Control group. ^b^ indicates a statistically significant difference (*p* < 0.05, Hedges’ *g* = −1.46) compared with the 3.5 GHz exposure group.

## Data Availability

All original contributions reported in this study are contained within the article. Further inquiries may be directed to the corresponding authors.

## Abbreviations

The following abbreviations are used in this manuscript:

5G	Fifth Generation
AR	Androgen Receptor
BCA	Bicinchoninic Acid
BSA	Bovine Serum Albumin
ECL	Enhanced Chemiluminescence
EL	Ejaculation latency
ELISA	Enzyme-linked immunosorbent assay
FCC	Federal Communication Commission
GHz	Gigahertz
HRP	Horseradish peroxidase
ICNIRP	International Commission on Non-Ionizing Radiation Protection
IF	Intromission frequency
KTX	Ketamine–Xylazine
MHz	Megahertz
mmWaves	Millimetre waves
MF	Mount frequency
ML	Mount latency
PBS	Phosphate-buffered saline
PEI	Post-ejaculatory interval
PMSF	Phenylmethylsulphonyl fluoride
PVDF	Polyvinylidene difluoride
RF-EMF	Radiofrequency electromagnetic field
RIPA	Radioimmunoprecipitation Assay
SDS-PAGE	Sodium Dodecyl Sulphate–Polyacrylamide Gel Electrophoresis
TBST	Tris-Buffered Saline with Tween-20
TMB	3′,5,5′-tetramethylbenzidine
WiFi	Wireless Fidelity
